# Water- and Nitrogen-Use Efficiencies of Hemp (*Cannabis sativa* L.) Based on Whole-Canopy Measurements and Modeling

**DOI:** 10.3389/fpls.2018.00951

**Published:** 2018-07-16

**Authors:** Kailei Tang, Alessandra Fracasso, Paul C. Struik, Xinyou Yin, Stefano Amaducci

**Affiliations:** ^1^Centre for Crop Systems Analysis, Plant Sciences, Wageningen University and Research, Wageningen, Netherlands; ^2^Department of Sustainable Crop Production, Università Cattolica del Sacro Cuore, Piacenza, Italy

**Keywords:** canopy gas exchange, hemp, *Cannabis sativa* L., nitrogen use efficiency, water use efficiency

## Abstract

Interest in hemp (*Cannabis sativa* L.) as a crop for the biobased economy is growing worldwide because hemp produces a high and valuable biomass while requiring low inputs. To understand the physiological basis of hemp's resource-use efficiency, canopy gas exchange was assessed using a chamber technique on canopies exposed to a range of nitrogen (N) and water levels. Since canopy transpiration and carbon assimilation were very sensitive to variations in microclimate among canopy chambers, observations were adjusted for microclimatic differences using a physiological canopy model, with leaf-level parameters estimated for hemp from our previous study. Canopy photosynthetic water-use efficiency (*PWUE*_c_), defined as the ratio of gross canopy photosynthesis to canopy transpiration, ranged from 4.0 mmol CO_2_ (mol H_2_O)^−1^ to 7.5 mmol CO_2_ (mol H_2_O)^−1^. Canopy photosynthetic nitrogen-use efficiency (*PNUE*_c_), the ratio of the gross canopy photosynthesis to canopy leaf-N content, ranged from 0.3 mol CO_2_ d^−1^ (g N)^−1^ to 0.7 mol CO_2_ d^−1^ (g N)^−1^. The effect of N-input levels on *PWUE*_c_ and *PNUE*_c_ was largely determined by the N effect on canopy size or leaf area index (*LAI*), whereas the effect of water-input levels differed between short- and long-term stresses. The effect of short-term water stress was reflected by stomatal regulation. The long-term stress increased leaf senescence, decreased *LAI* but retained total canopy N content; however, the increased average leaf-N could not compensate for the lost *LAI*, leading to a decreased *PNUE*_c_. Although hemp is known as a resource-use efficient crop, its final biomass yield and nitrogen use efficiency may be restricted by water limitation during growth. Our results also suggest that crop models should take stress-induced senescence into account in addition to stomatal effects if crops experience a prolonged water stress during growth.

## Introduction

The pressures of climate change, natural resource scarcity and environmental pollution have fuelled interest in bio-economically sustainable agronomy that requires effective use of scarcely available resources. A range of focused studies have indicated that hemp (*Cannabis sativa* L.) may be a suitable crop for the bio-economy (Amaducci and Gusovius, 2010). Hemp is a high-yielding multi-purpose crop that requires low inputs (Struik et al., 2000; Tang et al., 2016, 2017a) and has a positive impact on the environment (Bouloc and van der Werf, 2013; Barth and Carus, 2015). Its stems contain high-quality cellulose (De Meijer and van der Werf, 1994); high added-value compounds can be recovered from the female inflorescence and from threshing residues (Bertoli et al., 2010; Calzolari et al., 2017) after harvesting the seeds, that contain healthy oil (Leizer et al., 2000). Although once an important crop to produce raw materials for textiles and ropes, hemp acreage declined in the last century and was displaced largely by cotton and synthetic fibers. Consequently, little attention has been paid to understanding the physiological basis of the high resource-use efficiency of hemp.

Water and nitrogen deficiencies are major constraints in hemp production (Cosentino et al., 2013; Tang et al., 2017a). Focused quantitative studies on hemp's water- and nitrogen-use efficiencies are therefore needed. Crop water- and nitrogen-use efficiencies can be defined in different ways depending on the temporal and spatial scales of the processes and system aggregation they are based upon. The most important physiological process determining crop resource use efficiency is photosynthesis, at both leaf and canopy levels. With the aim of understanding the physiological basis of hemp's resource use efficiency, photosynthesis physiology of hemp was assessed in our previous study (Tang et al., 2017b) on leaves exposed to a range of nitrogen and temperature levels. Correlations between leaf photosynthesis and canopy photosynthesis are not always significant (Linderson et al., 2012; Tomás et al., 2012), because the latter is also affected by canopy size and the profile of resource distribution. The present study focuses on the scaling up of hemp photosynthesis from leaf to canopy and on analysing canopy photosynthetic water-use efficiency (*PWUE*_c_) and canopy photosynthetic nitrogen-use efficiency (*PNUE*_c_).

One challenge in studying *PWUE*_c_ and *PNUE*_c_ is to properly assess canopy CO_2_ and H_2_O exchange rates under varying nitrogen and water regimes. To date, the canopy gas exchange rate is mainly assessed by micro-meteorological methods or by means of canopy-enclosure chamber systems. The micro-meteorological techniques such as the eddy covariance or Bowen ratio methods enable gas flux measurements without disturbing canopy micro-environment, and they are often applied to large homogeneous areas but are unsuitable in plot/pot-sized experiments (Jones, 2013). In contrast, the canopy chamber technique enables to determine precisely canopy gas exchange at a relatively small scale (Müller et al., 2005, 2009). However, enclosing a crop canopy with a chamber might result in significant changes in micro-environmental variables (e.g., CO_2_ concentration, air temperature and vapor pressure) as a consequence of photosynthetic CO_2_ uptake, the greenhouse effect and transpiration (Takahashi et al., 2008; Müller et al., 2009). The effect of micro-environmental changes within a canopy chamber on photosynthesis rates should be assessed when the chamber is used to analyse the responses of canopy photosynthesis to water shortage and nitrogen deficiency.

On the basis of a thorough understanding of the underlying mechanisms of leaf and canopy photosynthesis, models have been developed to quantify the response of canopy photosynthesis to varying micro-environments under different physiological conditions (Hikosaka et al., 2016). Such canopy models are capable of simulating instantaneous canopy gas exchange measurements by micro-meteorological techniques (Leuning et al., 1998; Wright et al., 2013) and in canopy chambers (Müller et al., 2005). In that context, a well-defined canopy model is a useful tool to normalize the changes in micro-environmental variables within a canopy chamber and to quantitatively assess the responses of canopy photosynthesis to nitrogen and water deficiencies.

The objective of this study was to experimentally assess hemp *PWUE*_c_ and *PNUE*_c_ in relation to nitrogen and water availabilities. To that end, we parameterized a canopy photosynthesis model (Yin and van Laar, 2005; Yin and Struik, 2017), with leaf-level parameters estimated from our previous study for hemp (Tang et al., 2017b). This model was used for a dual-purpose: (i) to correct gas exchange measurements within different canopy chambers, and (ii) to assess the main components of hemp *PWUE*_c_ and *PNUE*_c_ in order to provide supporting information for efficient use of water and nitrogen resources.

## Materials and methods

### Experimental design and data collection

Field and container experiments were carried out at the research facilities of the Università Cattolica del Sacro Cuore (45.0° N, 9.8° E, 60 m asl; Piacenza, Italy). Field experiments were carried out in 2014 and 2015 to assess light and nitrogen distribution profiles of hemp canopies in response to nitrogen deficiency. A container experiment was carried out in 2014 to assess instantaneous and daily canopy gas exchange of hemp in response to nitrogen and water limitations. Between May and October (during the hemp season), the study site had monthly average temperatures ranging from 17.7 to 26.9°C; the monthly sum of precipitation ranged from 13.5 to 87.0 mm.

#### Field experiments to assess light and nitrogen distribution profiles of hemp canopies

The experimental fields had silty clay loam soil (the clay:silt:sand ratio was 39:46:15) that contained 0.14% of total nitrogen and 2.2–2.6% of organic matter. Seeds of hemp *cv*. Futura 75 (obtained from Fédération National des Producteurs de Chanvre, Le Mans, France) were drilled, with a target density of 120 plants m^−2^, at 3–4 cm depth using an experimental plot machine on 7 April in 2014 and on 16 April in 2015. Single plot size was 60 m^2^. Nutrients other than nitrogen were assumed to be abundantly available in the experimental fields based on past experience and analysis (data not shown). During the growth season, plants were irrigated when leaf angle distribution of the canopy became more erectophile during mid-day. A total of 60 mm and 155 mm water was provided with a traveling sprinkler in 2014 and 2015, respectively.

Nitrogen fertilization effect was investigated in a randomized complete block design with four replicates. In both years, four levels of calcium nitrate were top-dressed after seedling emergence as: N0 (no fertilizer applied); N30 (30 kg N ha^−1^); N60 (60 kg N ha^−1^), and N120 (120 kg N ha^−1^). In the field experiment in 2014, the plants suffered from severe weed competition. Therefore, only the data collected in the plots of N60 that were not affected by weeds were reported in this paper.

Two destructive samplings were conducted in each plot at the onset of the linear growth phase and at full flowering. At each sampling, light interception by the canopy (the ratio of light intensity at depth i to that at the top of canopy: *I*_i_/*I*_0_) was first assessed at 90, 75, 50, and 0% of canopy height using a ceptometer (AccuPAR LP-80, Decagon Devices, Inc., Pullman, Washington, USA). Subsequently, all plants in an area of 1 m^2^ were cut at ground surface to assess leaf area index (*LAI*) and specific leaf nitrogen (*SLN*) on four layers according to canopy height: 0–50, 50–75, 75–90, and 90–100%. The *LAI* was calculated as the product of leaf weight and specific leaf area (*SLA*) that was obtained by measuring the weight and area of all leaves of two representative plants. Leaf nitrogen concentration (*N*_leaf_) was assessed using a CN analyser (Vario Max CN Analyzer; Elementar Americas Inc., Hanau, Germany). The *SLN* was calculated as *N*_leaf_ divided by *SLA*.

#### Container experiment to assess canopy gas exchange rate

Seeds of *cv*. Futura 75 were sown on 9 May 2014 in 18 containers (length × width × height: 40 × 40 × 30 cm). Each container was filled with 23 kg of soil (dry weight) that contained 0.22% total nitrogen and had a clay:silt:sand ratio of 30:43:27. Seeds were sown in excess in two rows and seedlings were hand-thinned to 18 uniform plants per container (*ca*. 113 plants m^−2^). Other nutrients than nitrogen were assumed to be abundantly available based on past experience in the field from which the soil was collected. During the growth period, water was supplied daily to field capacity for each container. The containers were placed outdoor and positioned tightly in a 1.2 × 2.4 m block. To avoid any border effect, the block perimeter was surrounded with a green shading net (transmitting 3% of the light); the height of the shading net was adjusted daily to account for the increment in plant height. The containers were rearranged weekly.

Three levels of dissolved urea fertilizer were applied to the soil after seedling emergence as: N1, no fertilizer applied; N2, 1.0 g N per container; N3, 2.0 g N per container, equivalent to *ca*. 0, 60, and 120 kg N (ha ground)^−1^, respectively. There were six containers per N level, subject to different levels of water supply during measurement (see later).

Whole canopy gas exchange was assessed twice during the course of the experiment by enclosing the canopy of each container in a flow-through gas exchange system. The first cycle of measurements (CAN1 hereafter) aimed to assess the response of diurnal canopy gas exchange to nitrogen and short-term water shortage. Canopy gas exchange in this cycle was assessed on 12 containers for 3 days, four containers per N treatment. Two of the containers per N treatment were supplied with sufficient water (measured as the amount of transpired water in the previous day) during the measurement while the water supply for the other two was halved. This cycle of measurements started 49 days after sowing when the 6th−8th pair of leaves appeared, the same leaf stages at which gas exchange at leaf level was assessed (Tang et al., 2017b). The second cycle of canopy gas exchange assessment (CAN2 hereafter) aimed to assess the response of canopy gas exchange to prolonged water shortage. In this cycle, canopy gas exchange was assessed on six containers during 13 subsequent days, two containers per N treatment. Measurement in this cycle started 79 days after sowing at the beginning of flowering. During the measurement, one container received the amount of water transpired during the previous day while the other one received half the amount, with the exception of the 8th day from the start of measurement when plants under stress showed signs of severe wilting. At the 8th day from the start of measurements, the same amount of water was supplied to all containers to avoid possible death of the plants under stress before the end of the experimental period.

Configuration of the flow-through gas exchange system was described by Poni et al. (2014) and refined by Fracasso et al. (2017). It consists of 12 cylindrical canopy chambers (diameter 50 cm) that are sealed with flexible plastic polyethylene on the side wall (transmitting 87% of the light) and a plastic polymethylmethacrylate disc on the top (transmitting 93% of the light). The air flowing through the canopy chamber (from the bottom to the top) was drawn from 3 m above ground using two centrifugal blowers (Vorticent C25/2M, Vortice, Milan, Italy). The system records instantaneous information for each chamber every 12 min using a CR1000 datalogger wired to an AM16/32B Multiplexer (Campbell Scientific, Logan, USA) as follows: CO_2_ concentration, vapor pressure and air temperature at the entrance of the chamber (*CO*_2,in_, *VP*_in_, and *T*_in_, respectively) and the differences at the exit (*CO*_2,dif_, *VP*_dif_, and *T*_dif_, respectively; calculated as the value at exit minus that at entrance), container weight (*W*_container_) and incident solar radiation intensity outside the chamber. The *CO*_2,in_, *CO*_2,dif_, *VP*_in_ and *VP*_dif_ were assessed using a CIRAS-DC dual-channel absolute CO_2_/H_2_O infrared gas analyser (PP-Systems, Amesbury, USA). The *T*_in_ and *T*_dif_ were assessed using PFA-Teflon insulated type-T thermocouples (Omega Engineering, Stamford, USA). The *W*_container_ was monitored using a single cell platform scale placed under each container (ABC Bilance, Campogalliano, Italy).

In this study, the volume of each canopy chamber was 0.3 m^3^ (cross cutting area was 0.2 m^2^ and height was 1.5 m). Air flux entering each chamber was regulated at 4.3 × 10^−3^ m^3^ s^−1^. Thus, a complete volume air change required *ca*. 70 s. The flow rate was maintained constant during the whole measurement period. To prevent gas exchange between soil and plant chamber, the surface of each container was sealed with a plastic polyethylene film in which little slits were cut to allow hemp plants growing through. A small hole was made on the side wall of the container to supply water and allow gas exchange between soil and open air.

At the end of the canopy gas exchange assessment of each cycle, each container was assessed for the following parameters: the biomass weight of stems (*W*_stem_), green leaves (*W*_leaf,g_), senesced leaves (*W*_leaf,s_; if present), inflorescences (*W*_inflo_; if present), and roots (*W*_root_), *I*_i_*/I*_0_*, LAI* and *SLN*. For the containers receiving sufficient water in CAN1 the *I*_i_/*I*_0_, *LAI* and *SLN* were assessed for four layers according to canopy height: 0–50, 50–75, 75–90, and 90–100%, while for the remaining containers the same parameters were assessed on the entire canopy. To estimate any system error introduced by gas leakage or soil respiration, gas exchange measurements were performed for 1–2 days on each container after the plants had been cut.

### Data analysis

#### Estimation of light and nitrogen extinction coefficients

*PAR* was assumed to attenuate through the canopy following the Beer's law, based on *LAI*:

(1) $$\begin{array}{l} \frac{I_{\text{i}}}{I_{0}}=e^{-k_{\text{L}}LAI_{\text{i}}} \end{array}$$

where *LAI*_i_ is the *LAI* at depth i measured from the top; *k*_L_ is the light extinction coefficient. *k*_L_ was estimated by fitting the measured *I*_i_*/I*_0_ and *LAI*_i_ to Equation (1). To avoid any effect of measuring hour on the value of *k*_L_, all measured *I*_i_*/I*_0_ were normalized to a value at zenith angle 0°, according to the manufacturer manual of AccuPAR LP-80.

The vertical gradient of *SLN* can be similarly described (Yin et al., 2003; Archontoulis et al., 2011):

(2) $$\begin{array}{l} SLN_{\text{i}}=SLN_{0}e^{-k_{\text{n}}LAI_{\text{i}}} \end{array}$$

where *k*_n_ is the *SLN* extinction coefficient, *SLN*_0_ and *SLN*_i_ are the *SLN* at the top of the canopy (i.e., at *LAI*_i_ = 0) and at depth i, respectively. Thus, from canopy top to bottom, the cumulative nitrogen at depth i (*N*_i_) can be solved from Equation (2) as:

(3) $$\begin{array}{l} N_{\text{i}}=\int_{0}^{LAI_{\text{i}}}SLN_{\text{i }}\text{d}LAI_{\text{i}}=SLN_{0}\left(1-e^{-k_{\text{n}}LAI_{\text{i}}}\right)/k_{\text{n}} \end{array}$$

By fitting the measured data for *N*_i_-*LAI*_i_ relationships to Equation (3), *k*_n_ and *SLN*_0_ were estimated.

#### Calculation of canopy photosynthesis and transpiration rates

Data recorded from the multi-chamber gas exchange system was filtered to eliminate measurements impaired by short time fluctuations of air CO_2_ concentration and vapor pressure, and system mishaps. Subsequently, the values of *CO*_2,dif_ and *VP*_dif_ were corrected for potential system error due to gas leakage or soil respiration using data recorded in the chamber after the plants had been cut. Instantaneous canopy transpiration rate (*E*_c_; mmol H_2_O m^−2^ s^−1^) and net photosynthesis rate (*A*_c,net_; μmol CO_2_ m^−2^ s^−1^) were calculated using Equations (4, 5), respectively. These formulae were based on the study of Von Caemmerer and Farquhar (1981) for leaf gas exchange measurements. Different forms of these formulae were commonly used for calculating *E*_c_ and *A*_c,net_ in the studies of canopy gas exchange using the chamber system (Müller et al., 2005; Baker et al., 2009; Poni et al., 2014).

(4) $$\begin{array}{l} E_{\text{c}}=\frac{1000u_{\text{e}}VP_{\text{dif}}}{a\left[P-\left(VP_{\text{in}}+VP_{\text{dif}}\right)\right]} \end{array}$$

(5) $$\begin{array}{l} A_{\text{c},\text{ net}}=-\left(\frac{u_{\text{e}}CO_{2,\text{dif}}}{a}+{10^{-3}E}_{\text{c}}CO_{2,\text{ out}}\right) \end{array}$$

where *u*_e_ (mol s^−1^) is air flux entering the plant chamber; *a* (m^2^) is the ground area of the canopy chamber; *P* (kPa) is the air pressure inside the plant chamber. The standard air pressure (101.3 kPa) was used as a proxy of *P* in the present study although a slight overpressure was maintained inside the plant chamber (less than 10 Pa) to avoid any flux of ambient air through possible leaks. The effect of overpressure on *E*_c_ and *A*_c,net_ was considered negligible (Burkart et al., 2007).

Canopy gross photosynthesis (*A*_c,gross_) is the sum of *A*_c,net_ and canopy respiration (*R*_c_). *R*_c_ during the night was estimated directly from Equation (5) as *CO*_2,dif_ during the night was mainly a result of canopy respiration. During daytime, *R*_c_ was estimated considering the variation of temperature as:

(6) $$\begin{array}{l} R_{\text{c}}=R_{\text{c},25}\exp\left[\frac{E_{\text{Rc}}\left(T_{\text{air}}-25\right)}{298R\left(T_{\text{air}}+273\right)}\right] \end{array}$$

where *R*_c,25_ is the value of *R*_c_ at 25°C; *E*_Rc_ is the energy of activation; *R* is the universal gas constant (=8.314 J K^−1^ mol^−1^). The values of *R*_c25_ and *E*_Rc_ were estimated from the measurements of *R*_c_ during night (Reichstein et al., 2005).

#### Validation of a canopy photosynthetic model

The sun/shade model of De Pury and Farquhar (1997), as implemented in the crop model GECROS (Yin and van Laar, 2005; Yin and Struik, 2017), was validated against measured *A*_c,gross_. In this model, canopy leaves are divided into sunlit and shaded fractions and each fraction is modeled separately using a leaf photosynthesis model. When there is no water stress, potential leaf photosynthesis rate (*A*_p_) is calculated using an analytical solution of combined stomatal conductance, CO_2_ diffusion and biochemical leaf-photosynthesis models (Yin and Struik, 2009, 2017). In the presence of water limitation, actual available water for canopy transpiration (*E*_c_ in the present study) is considered as input to estimate actual stomatal resistance to water vapor (*r*_sw,a_) due to stomatal closure. The formula is expressed as (Yin and van Laar, 2005; Yin and Struik, 2017):

(7) $$\begin{array}{l} r_{\text{sw},\text{a}}=\left(E_{\text{p}}-E_{\text{a}}\right)\left(sr_{\text{bh}}+\gamma r_{\text{bw}}\right)/\left(\gamma E_{\text{a}}\right)+r_{\text{sw},\text{p}}E_{\text{p}}/E_{\text{a}} \end{array}$$

where *E*_a_ is actual available water for leaf transpiration while *E*_p_ is calculated from the Penman-Monteith equation representing potential leaf transpiration; *s* is the slope of the saturated vapor pressure curve; *r*_bh_, *r*_bw_, and *r*_sw,p_ are boundary layer resistances to heat, boundary resistance to water, and stomatal resistance to water transfer in absence of water stress, respectively; γ is the psychrometric constant (=0.067 kPa °C^−1^). For calculation of *s, r*_bh_ and *r*_bw_, see Yin and van Laar (2005) and Yin and Struik (2017). *r*_sw,p_ is assumed equal to 1/(1.6*g*_s_), where *g*_s_ is calculated according to *A*_p_. The estimated *r*_sw,a_ is then used to compute actual canopy photosynthesis in the presence of water limitation. Any non-stomatal effect of water stress on photosynthesis, which needs detailed biochemical modeling, is not considered in the present study. Relevant model algorithms are summarized in the Supplementary text.

The values of model input parameters required for leaf photosynthesis were presented in Tang et al. (2017b) for the same hemp cultivar and are summarized in Supplementary Table S1. The canopy related parameters *LAI, SLN, k*_L_ (for diffuse light) and *k*_n_ were derived in this study. The leaf angle that was used to calculate the direct light extinction coefficient was fixed at 15°, an average value assessed using a goniometer. Instantaneous environmental parameters, i.e., *CO*_2_, *VP, T*_air_ and irradiation intensity, were recorded by the canopy chamber system.

#### Normalization of gas exchange measurements within canopy chambers

The micro-environment differed between canopy chamber and ambient open air, and among treatments (see Results). Thus, the measured *E*_c_ and *A*_c,gross_ in the canopy chamber were normalized to that in the open air using the validated canopy model. Firstly, a correction factor *f*_Ec_ was obtained, based on simulated potential canopy transpiration *E*_cp_ as:

(8) $$\begin{array}{l} f_{\text{Ec}}=\frac{E_{\text{cp},\text{air}\left(\text{s}\right)}}{E_{\text{cp},\text{chamber}\left(\text{s}\right)}} \end{array}$$

where *E*_cp,air(s)_ and *E*_cp,chamber(s)_ are simulated potential canopy transpiration using weather data in open air and in the canopy chamber, respectively. The value of *E*_c_ corresponding to the open-air condition was then obtained by multiplying the measured *E*_c_ in the chamber with the correction factor *f*_Ec_. Subsequently, the corrected value of *E*_c_ for the open air and the measured *E*_c_ in the canopy chamber were used as inputs to obtain simulated canopy photosynthesis, *A*_c,gross, air(s)_ and *A*_c,gross, chamber(s)_, using weather data in the open air and in the canopy chamber, respectively. This gave a correction factor for *A*_c,gross_ (*f*_Ac_) as:

(9) $$\begin{array}{l} f_{\text{Ac}}=\frac{A_{\text{c},\text{gross},\text{ air}\left(\text{s}\right)}}{A_{\text{c},\text{gross},\text{chamber}\left(\text{s}\right)}} \end{array}$$

Finally, the value of *A*_c,gross_ corresponding to the open-air condition was calculated by multiplying the measured *A*_c,gross_ in the chamber with the factor *f*_Ac_.

#### Statistical analysis

Nonlinear fitting was carried out using the GAUSS method in PROC NLIN of SAS (SAS Institute Inc., Cary, NC, USA). Analysis of variance was conducted to assess the effects of nitrogen fertilization and water shortage on canopy structure and gas exchange related parameters using SPSS statistics 22.0 (SPSS, Chicago, Illinois, USA).

## Results

### The effects of nitrogen and water levels on canopy physiological parameters

Nitrogen fertilization resulted in an increase in canopy size and leaf nitrogen content. In the N60 plots where weed competition was negligible in the field experiment in 2014, *LAI* (leaf area index) was on average 3.2 and 4.8 m^2^ m^−2^ at linear growth stage and full flowering, respectively; *SLN* (specific leaf nitrogen) was on average 0.97 and 0.67 g N (m^2^ leaf)^−1^, respectively. In the field experiment in 2015, *LAI* of the N120 plots was 4.0 and 6.4 m^2^ m^−2^ at the onset of the linear growth stage and at full flowering, respectively, while *SLN* was 1.27 and 1.17 g N (m^2^ leaf)^−1^, respectively (Figure S1). Providing less nitrogen fertilization than 120 kg N ha^−1^ resulted in reductions in *LAI* and *SLN*. The *LAI* and *SLN* of N120 plots were on average 2.8 times and 1.2 times higher than those of non-fertilized canopies. In CAN1, *LAI* ranged from 1.8 to 2.6 m^2^ m^−2^; *SLN* ranged from 0.84 to 1.02 g N (m^2^ leaf)^−1^. Nitrogen fertilization in CAN1 resulted in increases in *LAI* and *SLN* by 40 and 19%, respectively (Table 1). For the well-watered containers in CAN2, the average values of *LAI* and *SLN* were 2.0 m^2^ m^−2^ and 0.68 g N (m^2^ leaf)^−1^, respectively (Table 2). Withholding water for 13 days in CAN2 resulted in an increase in the weight of senesced leaves while the weight of green leaves was reduced (Table 3). Consequently, water-stressed canopies had a 36% lower *LAI* than well-watered canopies (Table 2). While water stress resulted in a reduction in canopy size, the *SLN* of water-stressed canopies was 51% higher than that of well-watered canopies.

**Table 1:** The effects of nitrogen deficiency and short-term water shortage on canopy transpiration and carbon assimilation.

	***N*_C_**	***LAI***	***SLN***	***E*_c_**	***A*_c,gross_**	***PWUE*_c_**	***PNUE*_c_**
**NITROGEN**							
N1	1.53 b	1.84 b	0.84 b	162 b	0.70 b	4.49	0.46
N2	2.11 a	2.26 ab	0.94 ab	236 ab	0.94 ab	4.09	0.44
N3	2.58 a	2.59 a	1.02 a	268 a	1.14 a	4.41	0.44
*P-value*	0.00	0.00	0.03	0.03	0.02	0.32	0.78
**WATER**							
WS	2.08	2.36	0.88	187 b	0.84	4.65 a	0.40 b
WW	2.07	2.01	0.97	256 a	1.01	4.00 b	0.49 a
*P-value*	0.95	0.07	0.06	0.03	0.11	0.03	0.02

Data presented was collected in CAN1. The data of the last four columns is presented as the average of 3 days after being normalized to the open-air conditions. Canopy nitrogen content (N_c_; g N (m^2^ ground)^−1^), leaf area index (LAI; m^2^ m^−2^), specific leaf nitrogen (SLN; g N (m^2^ leaf)^−1^), actual transpiration (E_c_; mol H_2_O m^−2^ d^−1^), canopy gross photosynthesis (A_c,gross_; mol CO_2_ m^−2^ d^−1^), canopy photosynthetic water-use efficiency (PWUE_c_; mmol CO_2_ (mol H_2_O)^−1^) and canopy photosynthetic nitrogen-use efficiency (PNUE_c_; mol CO_2_ d^−1^ (g N)^−1^). N1, N2, and N3 denote nitrogen fertilization rate at 0, 1.0, and 2.0 g N container^−1^, respectively. WW denotes well-watered containers while WS denotes the containers where water supply was half of WW.

*ANOVA analysis was conducted considering nitrogen and water levels as main factors and measuring day as repeated factor. Interaction between nitrogen and water was excluded because it was not significant for all parameters in a preliminary test. Numbers followed by different letters under the same category are statistically different for P = 0.05 (Tukey HSD)*.

**Table 2:** The effects of nitrogen deficiency and long-term water shortage on canopy transpiration and carbon assimilation.

	***N*_C_**	***LAI***	***SLN***	***E*_c_**	***A*_c,gross_**	***PWUE*_c_**	***PNUE*_c_**
**NITROGEN**							
N1	0.79 b	1.15	0.71	69	0.36	6.28	0.44
N2	1.19 b	1.69	0.73	164	0.67	5.74	0.58
N3	2.08 a	1.99	1.12	182	0.87	5.58	0.42
*P*-value	0.024	0.06	0.19	0.39	0.36	0.72	0.61
**WATER**							
WS	1.35	1.26 b	1.03	43	0.31	7.53 a	0.26
WW	1.35	1.97 a	0.68	234	0.96	4.20 b	0.70
*P*-value	0.95	0.03	0.11	0.08	0.10	0.04	0.62

The data of the last four columns was collected in the last consecutive 3 days in CAN2 and is presented after being normalized to the open-air conditions.Canopy nitrogen content (N_c_; g N (m^2^ ground)^−1^), leaf area index (LAI; m^2^ m^−2^), specific leaf nitrogen (SLN; g N (m^2^ leaf)^−1^), actual transpiration (E_c_; mol H_2_O m^−2^ d^−1^), canopy gross photosynthesis (A_c,gross_; mol CO_2_ m^−2^ d^−1^), canopy photosynthetic water-use efficiency (PWUE_c_; mmol CO_2_ (mol H_2_O)^−1^) and canopy photosynthetic nitrogen-use efficiency (PNUE_c_; mol CO_2_ d^−1^ (g N)^−1^). N1, N2, and N3 denote nitrogen fertilization rate at 0, 1.0, and 2.0 g N container^−1^, respectively. WW denotes well-watered containers while WS denotes the containers where water supply was half of WW.

*ANOVA analysis was conducted considering nitrogen and water levels as main factors and measuring day as repeated factor. Interaction between nitrogen and water was excluded because it was not significant for all parameters in a preliminary test. Numbers followed by different letters under the same category are statistically different for P = 0.05 (Tukey HSD)*.

**Table 3:** The effects of long-term water shortage on the partitioning of biomass.

	**Biomass**	**Stem**	**Green leaf**	**Senesced leaf**	**Inflorescence**	**Root**
	**g m^−2^**	**g m^−2^**	**g m^−2^**	**g m^−2^**	**g m^−2^**	**g m^−2^**
WS	480	245	62.0	43.6	30.7	99
WW	590	278	96.9	37.3	49.0	128
*P-*value	0.10	0.23	0.04	0.05	0.08	0.22

Data presented was collected in CAN2.

WW denotes well-watered containers while WS denotes the containers where water supply was half of WW.

*Analysis of variance was performed considering canopy nitrogen content as covariate*.

Light intensity and *SLN* decreased progressively with increasing depth from top to bottom (Figure S2). The value of *k*_L_ (the light extinction coefficient) was 0.96 ± 0.04 m^2^ m^−2^ and was similar for nitrogen fertilization levels and growth environments (Figure S3A). The *SLN*_0_ (*SLN* at the top of the canopy) ranged from 1.43 to 2.72 g N (m^2^ leaf)^−1^ and the *k*_n_ (nitrogen extinction coefficient) ranged from 0.09 to 0.89 m^2^ m^−2^. The values of *k*_n_ decreased exponentially with an increase in *LAI* (Figure S3B). This relationship between *k*_n_ and *LAI* was consistent among nitrogen fertilization levels and growth environments. Thus, this relationship was applied to calculate *k*_n_ in subsequent model analyses.

### The effects of chamber system on canopy transpiration and photosynthesis

**Figure 1:**
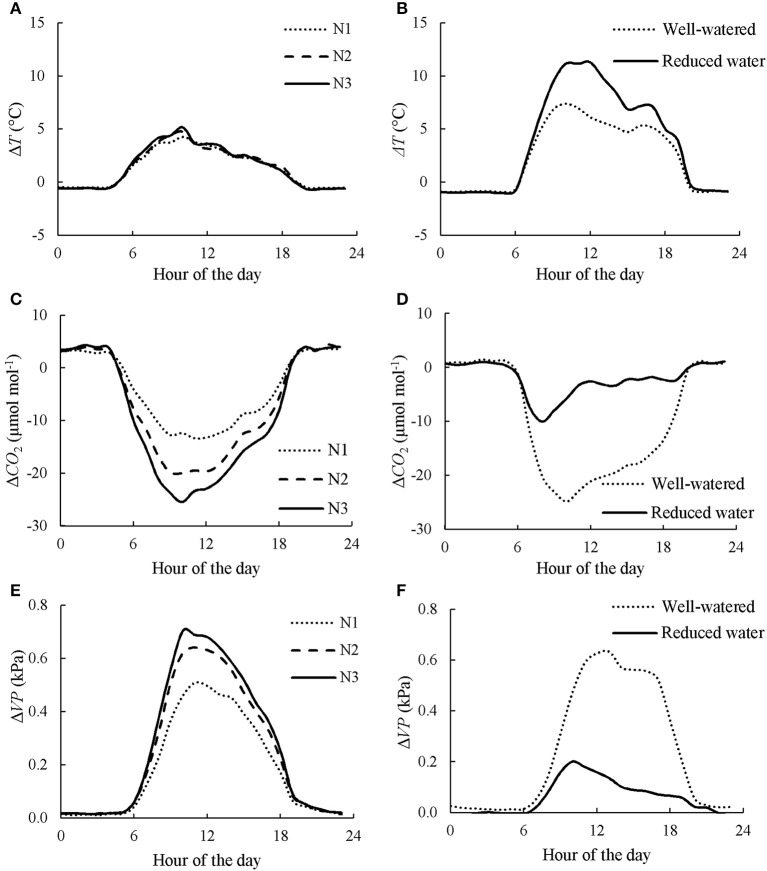
Diurnal courses of canopy chamber effects on air temperature (Δ*T*), CO_2_ concentration (ΔCO_2_), and vapor pressure (Δ*VP*) under different nitrogen **(A,C,E)** and water **(B,D,F)** regimes. Data presented in **A,C,E** is the average of 3 days in CAN1. N1, N2, and N3 denote the level of received nitrogen, see text for details. Data presented in **B,D,F** is the average of the last consecutive 3 days in CAN2.

The night-time chamber air temperature *T*_air_ ranged from 14.7 to 25.7°C and from 17.1 to 27.0°C during the measurements in CAN1 and CAN2, respectively. There was little difference in micro-environmental variables [i.e., *T*_air_, *CO*_2_ (CO_2_ concentration) and *VP* (vapor pressure)] during the night-time between chamber and ambient open air, and among treatments within chambers (Figure 1). During daytime, incident *PAR* reached up to 2,100 μmol m^−2^ s^−1^ while *T*_air_, *CO*_2_ and *VP* in the open air ranged from 17.6°C to 35.9°C, from 359.7 μmol mol^−1^ to 439.4 μmol mol^−1^, and from 1.7 kPa to 2.5 kPa, respectively. The daytime *T*_air_ and *VP* within chambers were higher than those in the open air while the *CO*_2_ was lower (Figure 1). Increasing nitrogen fertilization rate increased the differences in *T*_air_, *VP* and *CO*_2_ between chamber and ambient open air while reducing water supply increased the difference in *T*_air_ but decreased the differences in *VP* and *CO*_2_.

**Figure 2:**
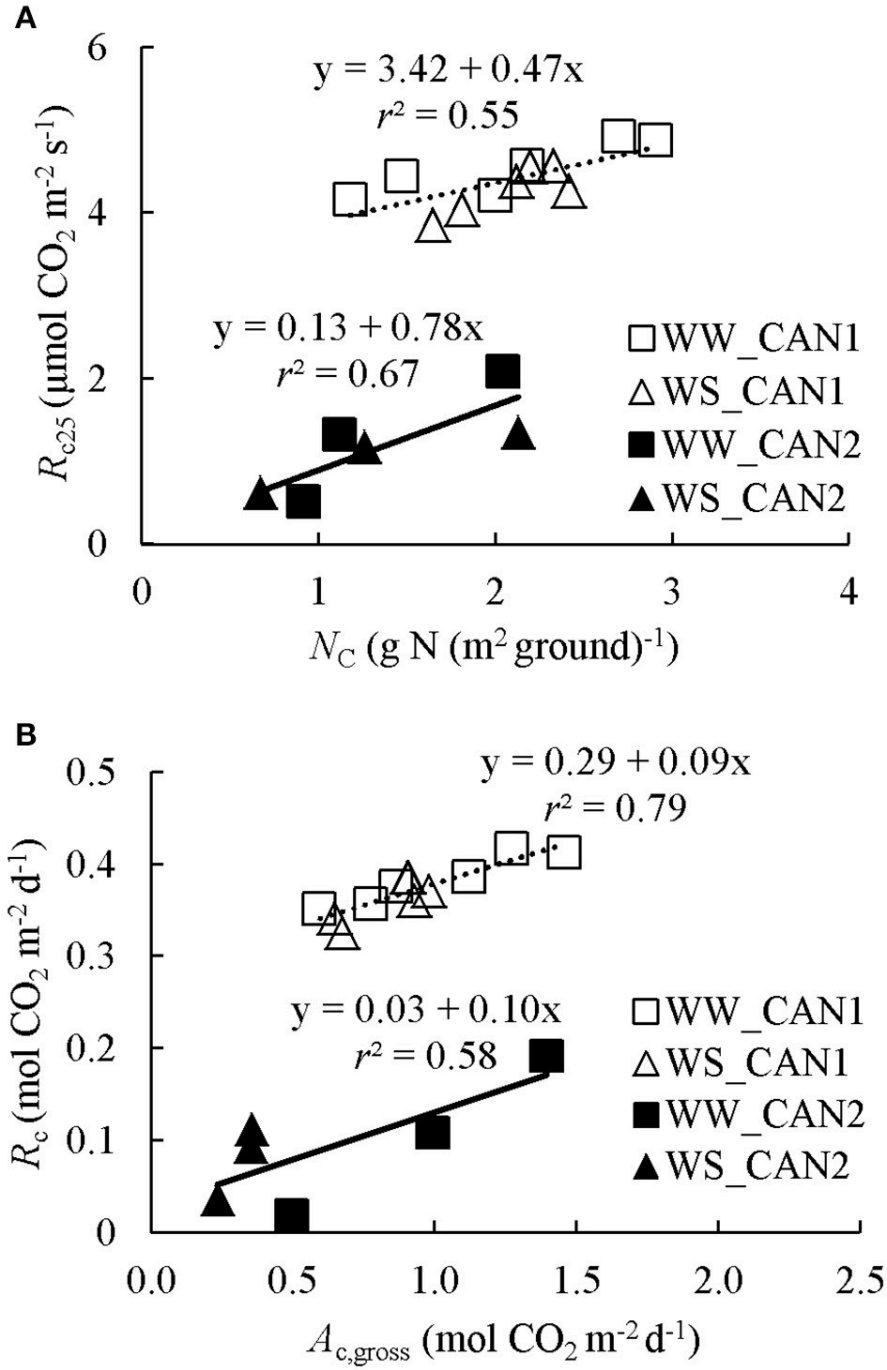
**(A)** Responses of canopy respiration at 25°C (*R*_c25_) to canopy leaf nitrogen content (*N*_C_). **(B)** Relationship between daily integrated canopy respiration (*R*_c_) and gross photosynthesis (*A*_c,gross_). WW and WS denote well-watered and water-limited conditions, respectively. CAN1 and CAN2 are experimental codes, see text for details.

The night-time canopy respiration *R*_c_ varied largely from minute to minute, presumably due to a relatively low *R*_c_ and high flow rate. Nevertheless, *R*_c_ increased slightly with increasing chamber *T*_air_ (Figure S4). By fitting these data to Equation (6), *E*_Rc_ (activation energy for *R*_c_) was estimated as 9,559 ± 2,779 J mol^−1^. The estimate of *R*_c25_ (*R*_c_ at 25°C) ranged from 3.9 to 4.9 μmol CO_2_ m^−2^ s^−1^ in CAN1, and from 0.50 to 2.09 μmol CO_2_ m^−2^ s^−1^ in CAN2 (Figure 2A). The difference in respiration rate between experiments was probably due to differences in growth stage. With the estimated *E*_Rc_ and *R*_c25_, instantaneous gross canopy photosynthesis rate *A*_c,gross_ in CAN1 and CAN2 was estimated. The daily *R*_c_ (canopy respiration) increased with increasing *A*_c,gross_ in both CAN1 and CAN2 but with different relationships (Figure 2B), and accounted for on average 40 and 15% of *A*_c,gross_ in CAN1 and CAN2, respectively.

**Figure 3:**
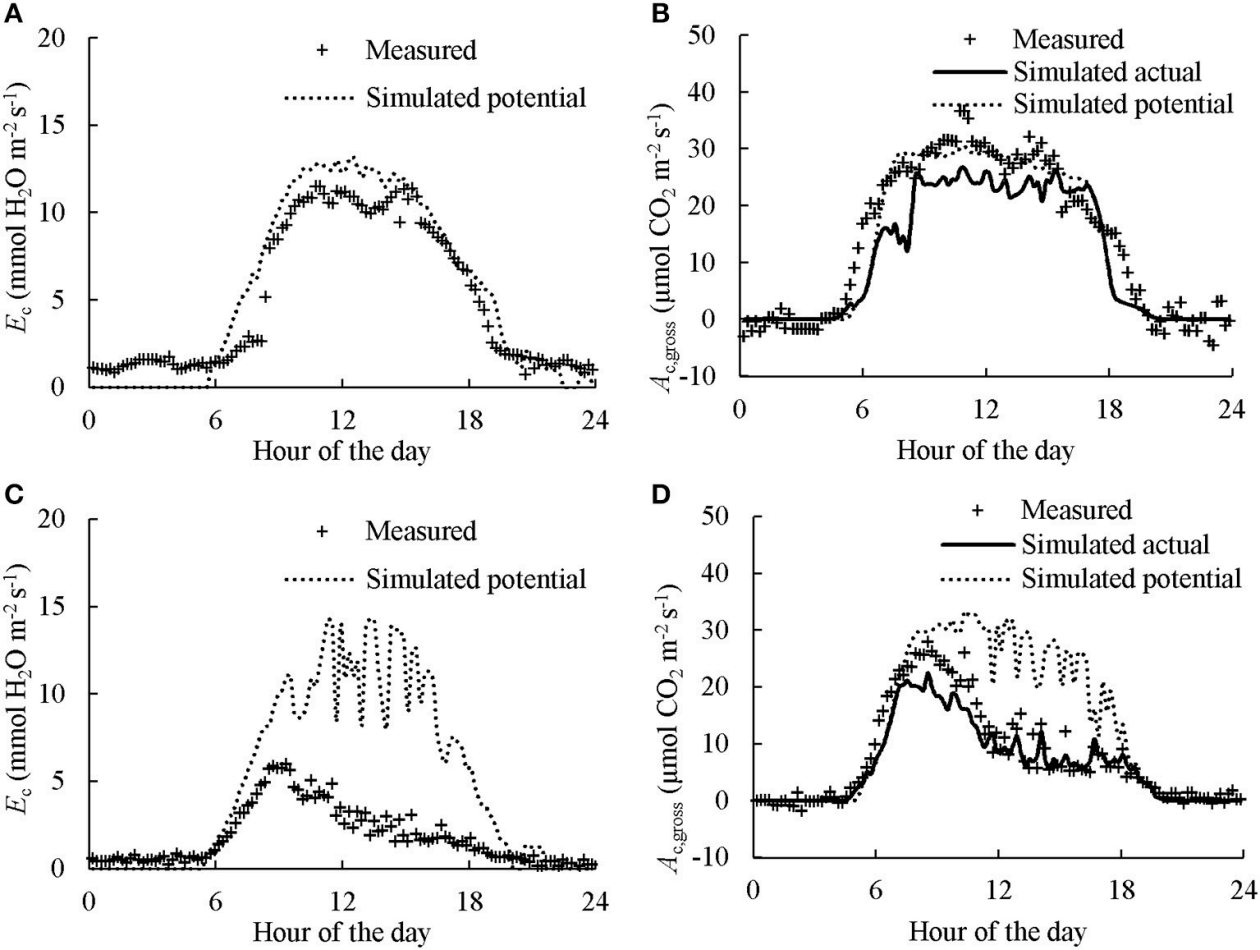
Diurnal courses of measured and simulated canopy transpiration (*E*_c_; **A,C**) and gross photosynthesis rates (*A*_c,gross_; **B,D**). The “Measured” dots present the values calculated from gas exchange measurement. The “Simulated potential” line presents the outcome of model simulation without considering water stress. The “Simulated actual” line in **B,D** presents the outcome of model simulation considering the estimated *E*_c_ as actual available water for transpiration while the effect of water deficiency on stomatal resistance was estimated using Equation (7). Data presented was collected in the first day of N2 in CAN1.The canopy in **A,B** received sufficient water while water supply in **C,D** was halved.

**Figure 4:**
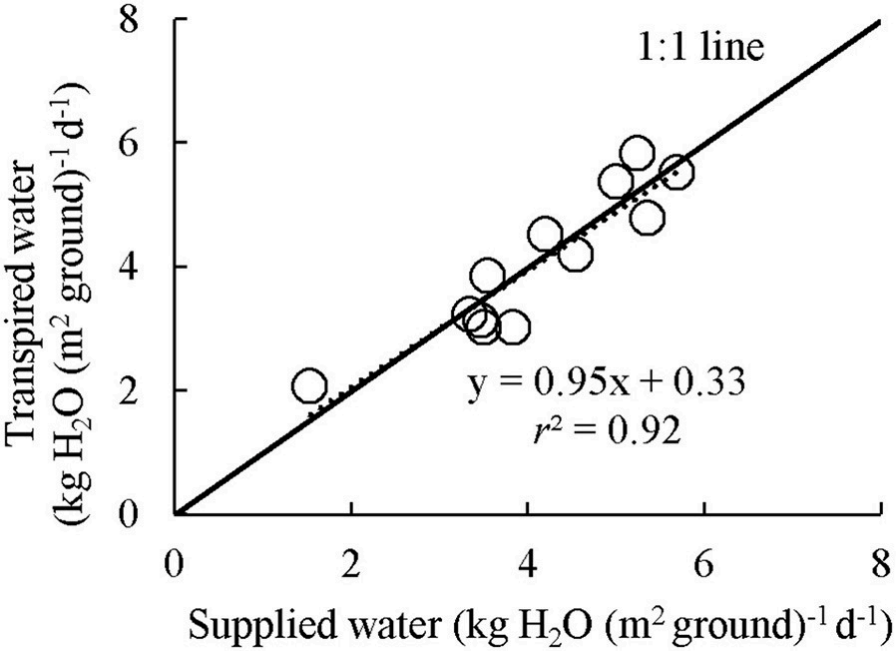
Integrated water loss through transpiration as measured by canopy gas exchange in comparison with the amount of supplied water. Each point represents the daily average of water loss versus water gain over the measuring period for each container in CAN1. The amount of supplied water was calculated as the difference of container weight at before and after watering.

Examples of diurnal courses of measured canopy transpiration *E*_c_ and *A*_c,gross_ within canopy chambers are presented in Figure 3. The *E*_c_ and *A*_c,gross_ were close to nil during night-time while during the daytime their values rose up to 11.1 mmol H_2_O m^−2^ s^−1^ and 38.1 μmol CO_2_ m^−2^ s^−1^, respectively. For the well-watered containers, the values of *E*_c_ and *A*_c,gross_ throughout the day followed closely their simulated potential transpiration *E*_cp_ and simulated potential photosynthesis *A*_cp,gross_ (Figure 3). As expected, the values of *E*_c_ and *A*_c,gross_ of the containers that received half amount of water were lower than their *E*_cp_ and *A*_cp,gross_ from the late morning to the end of daytime. Integration of the instantaneous *E*_c_ to daily values matched well with the amount of supplied water per day (Figure 4). Thus, the *A*_c,gross_ was simulated considering *E*_c_ as available water for transpiration at canopy level. The *E*_c_ was partitioned between sunlit and shaded leaves according to the relative share of their *E*_cp_ to obtain their actual transpiration (*E*_a_) at leaf level in Equation (7). There was a good agreement between the measured and simulated *A*_c,gross_ under different nitrogen and water regimes (Figure 3). The values of *r*^2^ and *rRMSE* for the comparison between measured and simulated values of all data points in CAN1 were 0.80 and 32%, respectively (part of the data points can be seen in Figure S5). For the measurements in CAN2, they were 0.78 and 66%, respectively.

The effects of micro-environmental differences between chamber and open air, and among treatments within chambers on canopy gas exchange were assessed using the validated model (Table 4). The presence of the plant chamber increased *E*_cp_ by 6.9–11.2% in CAN1 and by 19.6–34.2% in CAN2 while it decreased *A*_cp,gross_ by 0.3–1.4% in CAN1 and by 3.5–4.2% in CAN2. The chamber effect on *E*_cp_ varied little among nitrogen treatments while the effect on *A*_cp,gross_ increased with an increase in nitrogen rate. Water shortage increased the effects of the chamber on both *E*_cp_ and *A*_cp,gross_. Therefore, to account for any effect of varying micro-environmental variables due to the presence of the canopy chamber, the measured *E*_c_ and *A*_c,gross_ within each chamber were normalized to the conditions in the open air.

**Table 4:** The effects of plant chamber on canopy transpiration and photosynthesis under different nitrogen and water regimes.

	**CAN1**		**CAN2**	
	**Δ*E*_cp_ (%)**	**Δ*A*_cp,gross_ (%)**	**Δ*E*_cp_ (%)**	**Δ*A*_cp,gross_ (%)**
N1	9.0	−1.0	28.5	−3.5
N2	8.9	−1.0	25.5	−3.7
N3	9.1	−1.4	26.8	−4.2
WS	11.2	−1.2	34.2	−4.0
WW	6.9	−0.3	19.6	−3.6

Potential canopy transpiration (E_cp_) and photosynthesis (A_cp,gross_) were simulated using weather data in the open air and in the chambers for each treatment while the other parameters were kept at the average value of well-watered N3 containers. The differences of simulated E_cp_ and A_cp,gross_ between open air and plant chamber are presented as percentage of the value in the open air. The presence of the plant chamber resulted in an increase in E_cp_ while it resulted in a decrease in A_cp,gross_.

*N1, N2, and N3 denote nitrogen fertilization rate at 0, 1.0, and 2.0 g N container^−1^, respectively; WW denotes well-watered containers while WS denotes the containers where water supply was half of WW; CAN1 and CAN2 are experimental codes, see text for details*.

### The effects of nitrogen fertilization and short-term water shortage on canopy photosynthetic water- and nitrogen-use efficiencies

**Figure 5:**
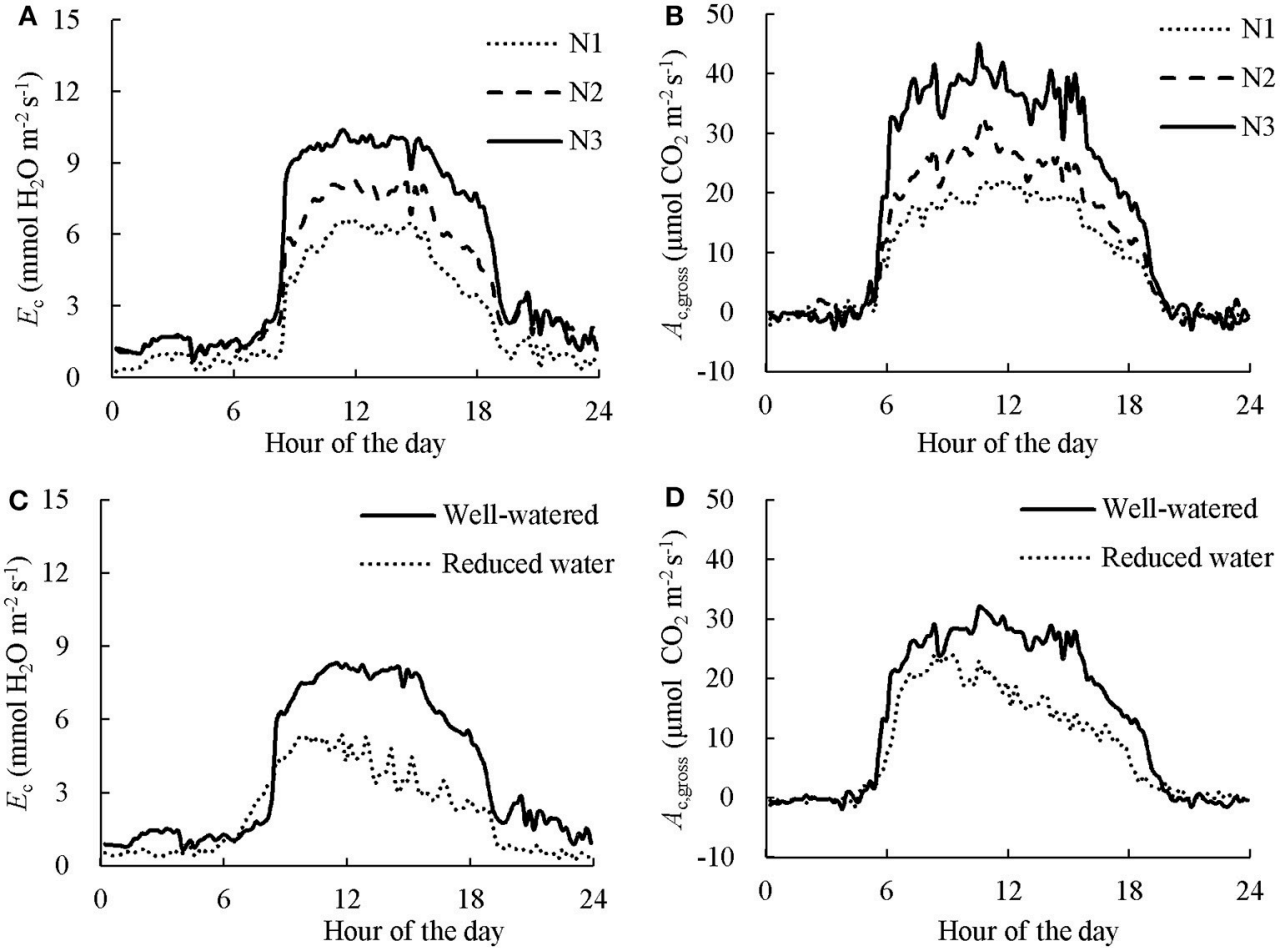
The effect of nitrogen **(A,B)** and short-term water stress **(C,D)** on instantaneous canopy transpiration **(A,C)** and photosynthesis rate **(B,D)**. Data presented was collected in the first day in CAN1. The data has been normalized to the open-air conditions. N1, N2, and N3 denote the level of received nitrogen, see text for details.

Examples of the diurnal courses of normalized *E*_c_ and *A*_c,gross_ in CAN1 are presented in Figure 5. Despite minute-to-minute fluctuations due to environmental variability, the *E*_c_ and *A*_c,gross_ were consistently higher in the fertilized canopies than in the non-fertilized canopies and water shortage resulted in reductions in *E*_c_ and *A*_c,gross_ occurring from the late morning to the end of the day. Consequently, daily integrated *E*_c_ and *A*_c,gross_ increased with an increase in nitrogen fertilization rate while they decreased under water limiting conditions (Table 1). The daily integrated *E*_c_ and *A*_c,gross_ ranged from 162 to 268 mol H_2_O m^−2^ d^−1^ and from 0.70 to 1.14 mol CO_2_ m^−2^ d^−1^, respectively. The canopy photosynthetic water-use efficiency (*PWUE*_c_), defined as the ratio of *A*_c,gross_ to *E*_c_, ranged from 4.00 to 4.65 mmol CO_2_ (mol H_2_O)^−1^. The *PWUE*_c_ did not differ significantly among nitrogen treatments while it increased by 16% under water limiting conditions compared to the control. The canopy photosynthetic nitrogen-use efficiency (*PNUE*_c_), the ratio of *A*_c,gross_ to *N*_c_, ranged from 0.40 to 0.49 mol CO_2_ d^−1^ (g N)^−1^. No significant effect of nitrogen fertilization on *PNUE*_c_ was observed (*P* > 0.05), while *PNUE*_c_ decreased significantly (by 18%; *P* < 0.05) under water limiting conditions (Table 1).

### The effects of long-term water shortage on canopy photosynthetic water- and nitrogen-use efficiencies

**Figure 6:**
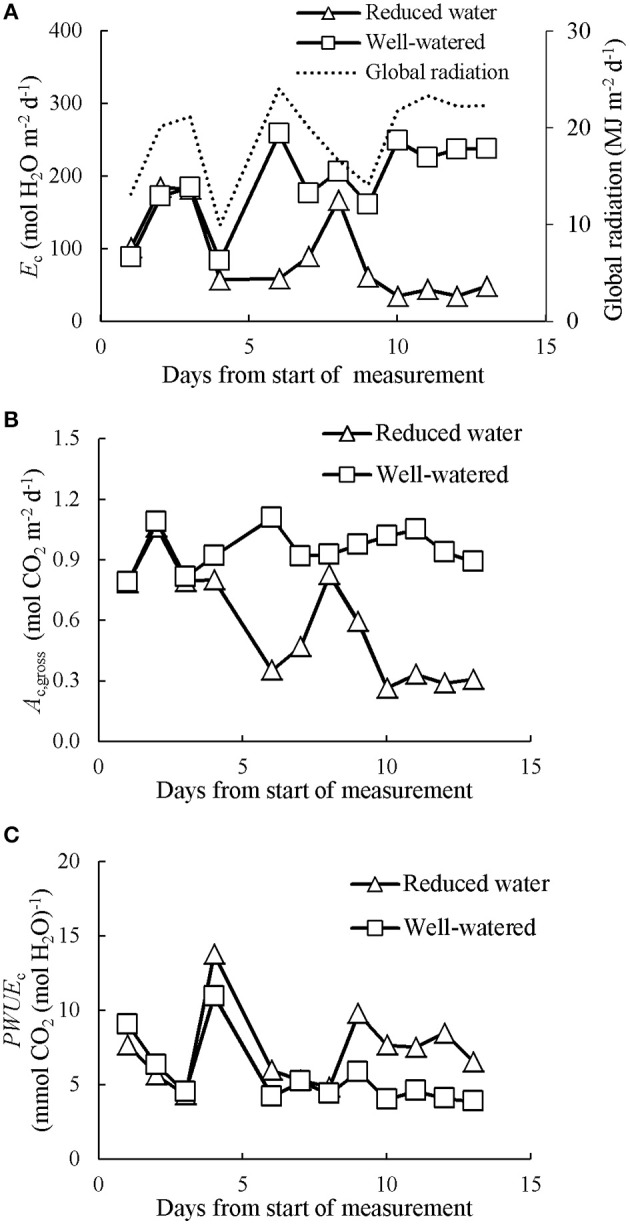
The evolution of prolonged water limitation effects on daily canopy transpiration (*E*_c_; **A**), gross photosynthesis (*A*_c,gross_; **B**) and canopy photosynthetic water-use efficiency (*PWUE*_c_; **C**). Data presented was collected in CAN2. The data has been normalized to the open-air conditions.

As water shortage was prolonged in CAN2, the progressive responses of *E*_c_, *A*_c,gross_, and *PWUE*_c_ are presented in Figure 6. Despite day to day fluctuations due to variable weather, reductions of *E*_c_ and *A*_c,gross_ emerged 4 days after withholding water and lasted until the end of the gas exchange measurements when all plants were cut for analysis. A short recovery was observed during the 8th day due to a brief re-watering of wilting plants in the water-stressed canopies (see Materials and Methods section). During the last 3 days, the average daily *E*_c_, *A*_c,gross_, *PWUE*_c_, and *PNUE*_c_ in the well-watered canopies were 234 mol H_2_O m^−2^ d^−1^, 0.96 mol CO_2_ m^−2^ d^−1^, 4.20 mmol CO_2_ (mol H_2_O)^−1^, and 0.70 mol CO_2_ d^−1^ (g N)^−1^, respectively (Table 2). The values of *E*_c_, *A*_c,gross_ and *PNUE*_c_ were higher than those of water-stressed canopies by 82, 68, and 63%, respectively, while the *PWUE*_c_ was lower than that of water-stressed canopies by 79%.

### The importance of canopy physiological parameters in determining canopy photosynthetic water- and nitrogen-use efficiencies

**Figure 7:**
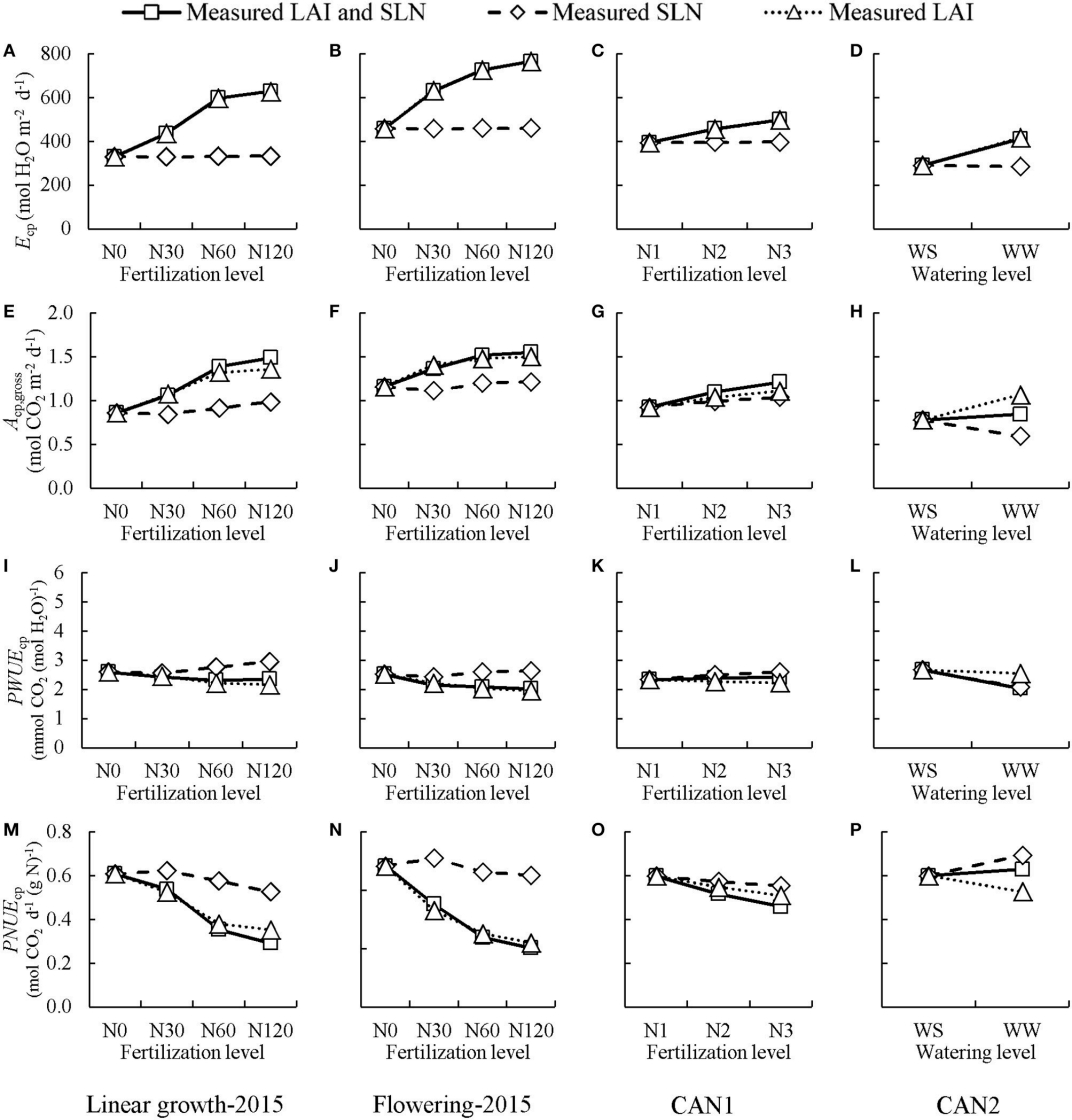
The simulated effects of nitrogen fertilization and water shortage on daily potential canopy transpiration (*E*_cp_; **A–D**), gross photosynthesis (*A*_cp,gross_; **E–H**), and canopy photosynthetic water-use efficiency (*PWUE*_cp_; **I–L**). and canopy photosynthetic nitrogen-use efficiency (*PNUE*_cp_; **M–P**). “Measured LAI and SLN” line: the default simulations performed using the measured leaf area index (*LAI*) and specific leaf nitrogen (*SLN*) at each nitrogen level or each water level. “Measured SLN” line: simulations performed with measured *SLN* at each nitrogen level (or each water level) while keeping *LAI* fixed at the values of non-fertilization (or water-stressed) treatment. “Measured LAI” line: simulations performed with measured *LAI* at each nitrogen level (or each water level) while keeping *SLN* fixed at the values of non-fertilization (or water-stressed) treatment. Linear growth-2015 **(A,E,I,M)** denotes the case where the values of *LAI* and *SLN* were collected at the linear growth stage in the field experiment in 2015; Flowering-2015 **(B,F,J,N)** denotes the case where the values of *LAI* and *SLN* were collected at full flowering in the field experiment in 2015; CAN1 **(C,G,K,O)** denotes the case where the values of *LAI* and *SLN* were collected in the CAN1; CAN2 **(D,H,L,P)** denotes the case where values of *LAI* and *SLN* were collected in the CAN2. N0, N30, N60, N120 denote nitrogen fertilization rate in 2015 at 0, 30, 60, and 120 kg N ha^−1^, respectively; N1, N2, and N3 denote nitrogen fertilization rate in CAN1 at 0, 1.0, and 2.0 g N container^−1^, respectively. Note the x-axes of the N treatment are not on scale. WW denotes well-watered containers in CAN2 while WS denotes the containers where water supply was half of WW.

Model analyses were performed to assess the relative importance of *LAI* and *SLN*, the two important canopy physiological parameters, in determining potential *PWUE*_c_ (*PWUE*_cp_) and *PNUE*_c_ (*PNUE*_cp_) in both the field experiment and the chamber experiment. This was done by first using the measured *SLN* and *LAI* of each nitrogen level as the default simulation and then forcing *LAI* or *SLN* of all treatments to their respective values at the non-fertilized or water stressed treatment (Figure 7). For both linear-growth and flowering stages of the field experiment, when forcing *SLN* to the value at non-fertilized treatment the values of *E*_cp_, *A*_cp,gross_, *PWUE*_cp_, and *PNUE*_cp_ changed little in comparison with those of the default simulation, whereas when forcing *LAI* to the value at non-fertilized treatment their values deviated significantly from the default simulation. In the chamber experiment in CAN1, the variations of *E*_cp_, *A*_cp,gross_, and *PWUE*_cp_ with increasing nitrogen rate were mainly due to a change in *LAI* whereas the variation of *PNUE*_cp_ was due to combined changes in *LAI* and *SLN*. In the chamber experiment in CAN2, the decrease in *E*_cp_ under long-term stress was mainly due to a change in *LAI* while the variations of *A*_c,gross_, *PWUE*_cp_, and *PNUE*_cp_ were due to combined changes in *LAI* and *SLN*.

## Discussion

Bio-economically sustainable agronomy requires effective use of scarce nitrogen and water resources. While hemp is considered as a bio-economically sustainable crop (Finnan and Styles, 2013; Amaducci et al., 2015; Tang et al., 2017b), its water- and nitrogen-use efficiencies have not been well addressed so far. As photosynthesis is the most important physiological process determining crop water- and nitrogen-use efficiencies, this study combined experimental and modeling analyses to assess the balance between canopy photosynthetic carbon gain and its water and nitrogen costs under different nitrogen and water regimes.

### Determination of canopy transpiration and photosynthesis

The canopy chamber technique is a useful tool to assess crop responses to nitrogen deficiency and water shortage at canopy scale. However, the presence of the chamber wall had a significant effect on the micro-environment within the chambers (Figure 1), confirming the results of previous studies (Poni et al., 1997; Takahashi et al., 2008; Müller et al., 2009). In the present study, a large difference of the micro-environment was also observed among nitrogen and water treatments that is probably due to their different rates of canopy transpiration and photosynthesis. The micro-environment conditions within the chamber resulted in a lower *A*_cp,gross_ and a higher *E*_cp_ than those in the open air, and this effect was larger in the chambers with higher fertilization rate and lower water supply (Table 4). As responses of *E*_c_ and *A*_c,gross_ to environmental variables are probably not linear (Hikosaka et al., 2016), it is necessary to normalize measurements within different chambers to avoid any confounding effect due to the differences in chamber micro-environmental factors.

In line with previous studies (Leuning et al., 1998; Müller et al., 2005), the variation of *E*_c_ and *A*_c,gross_ in response to fluctuating environmental conditions under different nitrogen and water regimes can be precisely described using a process-based physiological model (Figure 3). Thus, discrepancies in *E*_c_ and *A*_c,gross_ among chambers due to differences in micro-environment at measuring time could be properly accounted for through correction factors *f*_Ec_ and *f*_Ac_, respectively (see Equations 8, 9), in our study.

### Hemp canopy photosynthetic water- and nitrogen-use efficiencies in relation to nitrogen availability

The reason for the lack of significant responses of *PWUE*_c_ and *PNUE*_c_ to the decrease in nitrogen rate in the container experiments is not clear (Table 1). It is probably due to small variations in *LAI* and *SLN* among nitrogen treatments. This is confirmed in the model analysis for the field experiment in 2015, where the variation in *LAI* among N treatments was much more significant than that in our container experiment. This model analysis suggested that both *PWUE*_c_ and *PNUE*_c_ increased with decreasing nitrogen fertilization rate, and that the increases in *PWUE*_c_ and *PNUE*_c_ were mainly a result of a reduction in *LAI* (Figure 7). The reduced *LAI* resulted in increases in *PWUE*_c_ and *PNUE*_c_, i.e., the reduction in *A*_c,gross_ with a decrease in *LAI* is less than the reductions in *E*_c_ and in *N*_C_. This could be explained by an optimum *SLN* gradient relative to the light gradient in the canopy. It has been reported that the profile of *SLN* in a canopy is a whole-plant process that depends on canopy size (Moreau et al., 2012). Our data showed that the value of *k*_n_ increased with decreasing *LAI* (Figure S3B), up to a value of *ca*. 0.9 close to the LAI-independent value of *k*_L_ (0.96 m^2^ m^−2^, Figure S3A). So, the value of *k*_n_ in a large hemp canopy was generally lower than its theoretical value for a maximized canopy photosynthesis, which could be achieved only when *k*_n_ = *k*_L_ (Hirose and Werger, 1987; Hikosaka et al., 2016). When *LAI* is low, canopy photosynthesis is close to a maximum value as a result of *k*_n_ being close to *k*_L_; in such a case, the average leaf photosynthesis rate could be increased for a given amount of *N*_c_, while *E*_c_ stayed largely unchanged.

The variation in *PWUE*_c_ and *PNUE*_c_ with decreasing nitrogen fertilization rate may also be attributed to the variation in the absolute amount of *SLN*. It has been widely reported that *SLN* positively correlates with water-use efficiency while it negatively correlates with nitrogen-use efficiency at leaf level (Van den Boogaard et al., 1995; Shangguan et al., 2000; Cabrera-Bosquet et al., 2007). However, in response to nitrogen stress, hemp tends to maintain *SLN* at the expense of *LAI* (Figure S1). This response is in line with that of sunflower (*Helianthus annuus* L.), canola (*Brassica napus* L.) and wheat (*Triticum aestivum* L.) whereas it contrasts with that of maize (*Zea mays* L.), which tends to maintain *LAI* under nitrogen stress at the expense of *SLN* (Lemaire et al., 2008). As a result of the relative small variation in *SLN* among nitrogen treatments, little effect of *SLN* was detected on the hemp *PWUE*_c_ and *PNUE*_c_ (Figure 7).

The effects of nitrogen fertilization on crop water-use efficiency and nitrogen-use efficiency are whole plant processes that depend on leaf photosynthetic capacity and canopy size, and our analysis showed that relative to leaf photosynthetic capacity (determined by *SLN*), canopy size (*LAI*) plays a predominant role in this.

### Hemp canopy photosynthetic water- and nitrogen-use efficiencies in relation to water availability

Field observations generally show that water stress results in an increase in hemp water-use efficiency (Cosentino et al., 2013). This is confirmed by our results showing both short-term and long-term water shortages that resulted in an increase in *PWUE*_c_ (Tables 1, 2). However, our study further showed that the effect differed between short- and long-term stresses.

In response to short-term water stress, the increase in *PWUE*_c_ is mainly a consequence of stomatal closure as the variations in *E*_c_ and *A*_c,gross_ with decreasing water supply were precisely captured by considering the response of stomatal conductance (Figure 3). In fact, stomatal closure is one of the earliest responses to water deficit, protecting the plants from extensive water loss (Chaves et al., 2003). Stomatal closure restricts both H_2_O and CO_2_ exchange between leaf intercellular and ambient air that leads to great decreases in *E*_c_ and *A*_c,gross_ (Table 1; Figure 3). However, the reductions in *E*_c_ and *A*_c,gross_ are not parallel and the *PWUE*_c_ increased under water stress, probably because of the non-linear relationship between carbon assimilation rate and CO_2_ concentration in the intercellular space (Tang et al., 2017b). The higher value of *PWUE*_c_ under water stress indicates that the estimation of canopy photosynthesis under water limiting condition by assuming a consistent *PWUE*_c_ in the crop models, such as SUCROS (van Laar et al., 1997), is only an approximation. Instead, the present study considered the response of stomatal conductance using Equation (7) that results in higher value of *PWUE*_c_ under water stress. This approach is therefore preferable in the simulation of canopy photosynthesis under short-term water stress conditions. Nevertheless, we could not exclude the possibility that non-stomatal limitations were involved in our experiment. Further researches are needed to understand the effect of non-stomatal change under water-stress conditions on canopy photosynthesis, such as change of leaf angle (Archontoulis et al., 2011).

As water stress continued, hemp responded through reducing *LAI* and increasing *SLN*, while *N*_C_ stayed unchanged (Table 2). The reduced *LAI* was largely caused by increased senescence (Table 3). Because of this additional response, model analysis for the sensitivity in response to changing *LAI* or *SLN* was contrasting between CAN1 and CAN2 (Figure 7). This type of response to a long-term water stress was also observed in studies on other species such as kenaf (*Hibiscus cannabinus*) and sunflower (Danalatos and Archontoulis, 2010; Archontoulis et al., 2011). The response could result in more significant increases in *PWUE*_c_ as a result of both the stomatal response discussed above and the reduced evaporative surfaces. However, an increase in *SLN* could not compensate for the loss in *LAI*; so, the long-term stress resulted in large reductions in the *A*_cp,gross_, *E*_cp_, and *PNUE*_c_ (Table 2). This result indicates that crop models for predicting the effect of long-term water stress should introduce mechanisms on the responses of canopy-level traits (like *LAI*) in addition to stomatal regulation. It also suggests that although hemp is tolerant to long-term water stress through improving water-use efficiency (Cosentino et al., 2013), its final biomass yield and nitrogen-use efficiency may be restricted largely by water limitation during growth.

## Author contributions

KT, XY, and SA conceived and planned the experiment. KT and AF set up and monitored the canopy gas exchange system. KT performed the experimental work and data analysis. KT and AF drafted the manuscript. SA, PS, and XY revised the manuscript. SA and XY supervised the research. All the authors have read and approved the final manuscript.

### Conflict of interest statement

The authors declare that the research was conducted in the absence of any commercial or financial relationships that could be construed as a potential conflict of interest.
