# Supplementary material for: Water- and Nitrogen-Use Efficiencies of Hemp (Cannabis sativa L.) Based on Whole-Canopy Measurements and Modeling
Source: Front Plant Sci. 2018 Jul 16;9:951. doi: 10.3389/fpls.2018.00951 (PMC6055055; doi:10.3389/fpls.2018.00951)
Supplement: Supplementary file 1 [file Presentation_1.PDF]

Supplementary Text Summary information for the models used in this study

Leaf photosynthesis model The model of Farquhar, von Caemmerer & Berry (1980; the FvCB model hereafter) calculates net CO_2-assimilation rate (A) as the minimum of the Rubisco-limited (A_c), electron (e^-) transport-limited (A_j), and triose phosphate utilisation-limited (A_p) rates. The three limiting rates can be expressed collectively as:

$$A = \frac{(C_c - \Gamma_*)x_1}{C_c + x_2} - R_d \quad \text{(A1)}$$

where for A_c: $x_1 = V_{cmax}$ and $x_2 = K_{mC}(1+O/K_{mO})$; for A_j: $x_1 = J/4$ and $x_2 = 2\Gamma_*$; and for A_p: $x_1 = 3T_p$ and $x_2 = -\Gamma_*$. In the model, C_c and O are the CO_2 and O_2 level, respectively, at the carboxylation sites of Rubisco, Γ_* is the CO_2 compensation point in the absence of day respiration (R_d), and J is the linear e^- transport rate and is described as a function of incident irradiance I_{inc} as:

$$J = \left(\kappa_{2LL}I_{inc} + J_{max} - \sqrt{(\kappa_{2LL}I_{inc} + J_{max})^2 - 4\theta J_{max}\kappa_{2LL}I_{inc}}\right)/(2\theta) \quad \text{(A2)}$$

The sub-model for stomatal conductance for CO_2 transfer (g_s) is:

$$g_s = g_0 + \frac{A + R_d}{C_i - C_{i*}} f_{vpd} \quad \text{(A3)}$$

where g_0 is the residual value of g_s when irradiance approaches to zero, C_{i*} is the intercellular CO_2 level (C_i) at which $A+R_d = 0$, and f_{vpd} is the relative effect of the leaf-to-air vapour difference (VPD) on g_s (see later).

CO_2 transfer from C_a (the ambient CO_2 level) to C_c can be written as (Flexas et al. 2013):

$$C_i = C_a - A(1/g_b + 1/g_s) \quad \text{(A4)}$$

$$C_c = C_i - A/g_m \quad \text{(A5)}$$

Combining eqns (A1, A3-A5) gives a standard cubic equation for solution to A. The solution is complicated and not shown here but see Yin & Struik (2009; 2017).

In the g_s model, Eqn (A3), f_{vpd} is the function for the effect of VPD, which may be described phenomenologically as (Yin & Struik 2009):

$$f_{vpd} = \frac{1}{1/(a_1 - b_1 \cdot VPD) - 1} \quad \text{(A6)}$$

where a_1 and b_1 represent the C_i:C_a ratio in water vapour saturated air and the slope of the decrease of this ratio with increasing VPD, respectively, if g_0 approaches nil.

A number of parameters are related to leaf temperature (T_l), and some of these can be described by the Arrhenius equation normalised with respect to 25°C:

$$\text{Parameter} = \text{Parameter}_{25} \cdot e^{\left(\frac{1}{298}-\frac{1}{273+T_l}\right)\frac{E}{R}} \quad \text{(A7)}$$

where R is the universal gas constant (8.314 J K^{-1} mol^{-1}). Eqn (A7) applies to R_d, Γ_*, V_{cmax}, K_{mC}, K_{mO}, T_p, and b_1. The temperature response of J_{max} is described by the modified Arrhenius equation:

$$\text{Parameter} = \text{Parameter}_{25} \cdot e^{\left(\frac{1}{298}-\frac{1}{273+T_l}\right)\frac{E}{R}} \cdot \frac{1+e^{(S-D/298)/R}}{1+e^{[S-D/(273+T_l)]/R}} \quad \text{(A8)}$$

The values at 25°C of parameters R_d, V_{cmax}, J_{max}, and T_p can be further quantified as a linear function of specific leaf nitrogen (SLN) above a certain base value (n_b), at or below which leaf photosynthesis is zero:

$$\text{Parameter}_{25} = \chi(SLN - n_b) \quad \text{(A9)}$$

where χ have different values for different parameters.

All these parameter values were based on our previous estimate for hemp (Tang et al. 2017b) or based on the literature for those conservative parameter values in C_3 species (see Table S1).

Leaf transpiration model When there is no water stress, photosynthesis rate largely determines the transpiration rate. The basic equation to estimate potential leaf transpiration, E_p, is the Penman-Monteith equation (Monteith 1973):

$$E_p = \frac{sR_n + \rho c_p D_a / r_{bh}}{\lambda\{s + \gamma[(r_{bw} + r_{sw,p})/r_{bh}]\}} \quad \text{(A10)}$$

where R_n is net absorbed radiation, r_{bh} and r_{bw} are the boundary layer resistance to heat and water transfer, respectively, $r_{sw,p}$ is the stomatal resistance to water transfer if there is no water stress, D_a is saturation vapour pressure deficit of the external air, ρc_p is volumetric heat capacity of air, λ is the latent heat of vapourisation of water, γ is the psychrometric constant. Calculation of r_{bw}, r_{bh}, and R_n was the same as used in the GECROS model (Yin & Struik 2017).

In the presence of water limitation, actual transpiration is assumed to be the amount of actual available water. Then, the change of actual stomatal resistance to water vapour ($r_{sw,a}$) due to stomatal closure was obtained (see Eqn 7 in the main text), and this actual $r_{sw,a}$ was then used

to calculate actual photosynthesis using an analytical, quadratic solution as presented by Yin & Struik 2017).

Scaling up to canopy The sun/shade model (de Pury & Farquhar 1997) is adopted, in which the canopy is divided into sunlit and shaded fractions and each fraction is modelled separately with a single-layer leaf model (described above).

Radiation absorbed by a canopy, I_c, was determined as:

$$I_{c} = (1-\rho_{cb})I_{b0}(1-e^{-k_b' L}) + (1-\rho_{cd})I_{d0}(1-e^{-k_d' L}) \tag{A11}$$

where I_{b0} and I_{d0} are incident direct-beam and diffuse radiation above the canopy, ρ_{cb} and ρ_{cd} are canopy reflection coefficient for direct-beam and diffuse light, respectively, k_b' and k_d' are extinction coefficients for beam and scattered beam, diffuse and scattered diffuse lights, respectively.

Radiation absorbed by the sunlit fraction of the canopy, $I_{c,su}$, is given as the sum of direct-beam, diffuse, and scattered beam components (de Pury & Farquhar 1997):

$$\begin{aligned} I_{c,su} &= (1-\sigma)I_{b0}(1-e^{-k_b L}) + (1-\rho_{cd})I_{d0}\frac{k_d'[1-e^{-(k_d'+k_b)L}]}{k_d'+k_b} \\ &+ I_{b0}\left\{(1-\rho_{cb})\frac{k_b'[1-e^{-(k_b'+k_b)L}]}{k_b'+k_b} - (1-\sigma)\frac{1-e^{-2k_b L}}{2}\right\} \end{aligned} \tag{A12}$$

where σ is leaf scattering coefficient.

Radiation absorbed by the shaded fraction of the canopy, $I_{c,sh}$, is calculated as the difference between the total radiation absorbed by the canopy, I_c, and the radiation absorbed by the sunlit fraction, $I_{c,su}$ (de Pury & Farquhar 1997):

$$I_{c,sh} = I_c - I_{c,su} \tag{A13}$$

Eqns (A11-A13) were applied separately to visible or photosynthetically active radiation (PAR) and near-infrared radiation (NIR), because they have different values for σ, ρ_{cb}, ρ_{cd}, k_b, k_b' and k_d'. The model assumes that half of the incident solar radiation is in the visible and other half is in the NIR waveband. All these coefficients are described as in GECROS (Yin & Struik 2017).

Many photosynthetic parameters are related to *SLN*, and r_{bh} and r_{bw} are related to wind speed *u*. Both *SLN* and *u* change with the depth of the canopy. To estimate these parameters for the entire canopy, and for the sunlit and shaded fractions of the canopy, photosynthetically

active leaf nitrogen has to be scaled up. Assuming an exponential profile for the vertical decline of *SLN* in the canopy (Figure S1), photosynthetically active nitrogen for the entire canopy (N_{cp}), for the sunlit fraction of the canopy ($N_{cp,su}$) and for the shaded fraction of the canopy ($N_{cp,sh}$), can be estimated by (Yin & van Laar 2005):

$$N_{cp} = SLN_0(1-e^{-k_n L})/k_n - n_b L \quad \text{(A14)}$$

$$N_{cp,su} = SLN_0[1-e^{-(k_n+k_b)L}]/(k_n+k_b) - n_b(1-e^{-k_b L})/k_b \quad \text{(A15)}$$

$$N_{cp,sh} = N_{cp} - N_{cp,su} \quad \text{(A16)}$$

where SLN_0 is the SLN for uppermost leaves, k_n is the leaf nitrogen extinction coefficient in the canopy (see the main text). With a similar logic, boundary-layer conductance can be scaled up to the canopy level.

References used in Supplementary Text and Table S1 not already cited in main text

Bernacchi C.J., Portis A.R., Nakano H., von Caemmerer S. and Long S.P. (2002) Temperature response of mesophyll conductance. Implications for the determination of Rubisco enzyme kinetics and for limitations to photosynthesis in vivo. Plant Physiology, 130, 1992-1998.

de Pury D. and Farquhar G. (1997) Simple scaling of photosynthesis from leaves to canopies without the errors of big-leaf models. Plant, Cell and Environment, 20, 537-557.

Farquhar G.D., von Caemmerer S. & Berry J.A. (1980) A biochemical model of photosynthetic CO_2 assimilation in leaves of C_3 species. Planta, 149, 78-90.

Flexas J., Niinemets Ü., Gallé A., Barbour M.M., Centritto M., Diaz-Espejo A., Douthe C., Galmés J., Ribas-Carbo M. & Rodriguez P.L. (2013) Diffusional conductances to CO_2 as a target for increasing photosynthesis and photosynthetic water-use efficiency. Photosynthesis research, 117, 45-59.

Monteith, J.L. 1973. Principles of environmental physics. Edward Arnold, London.

Ögren E. & Evans J. (1993) Photosynthetic light-response curves. Planta, 189, 182-190.

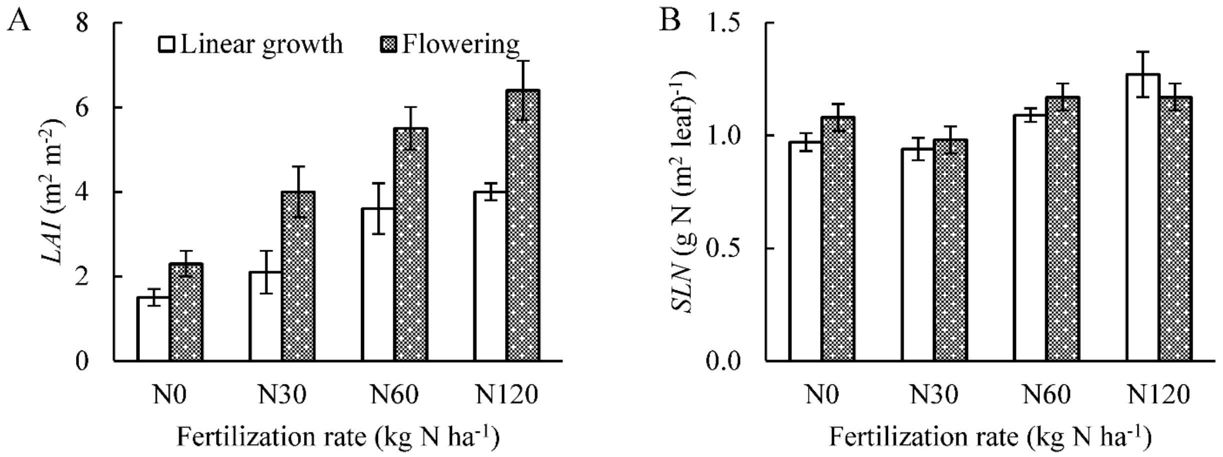

Supplementary Figure S1. The effects of nitrogen fertilization on leaf area index (*LAI*; Panel A) and specific leaf nitrogen (*SLN*; Panel B) at the onset of linear growth and at full flowering in the field experiment in 2015.

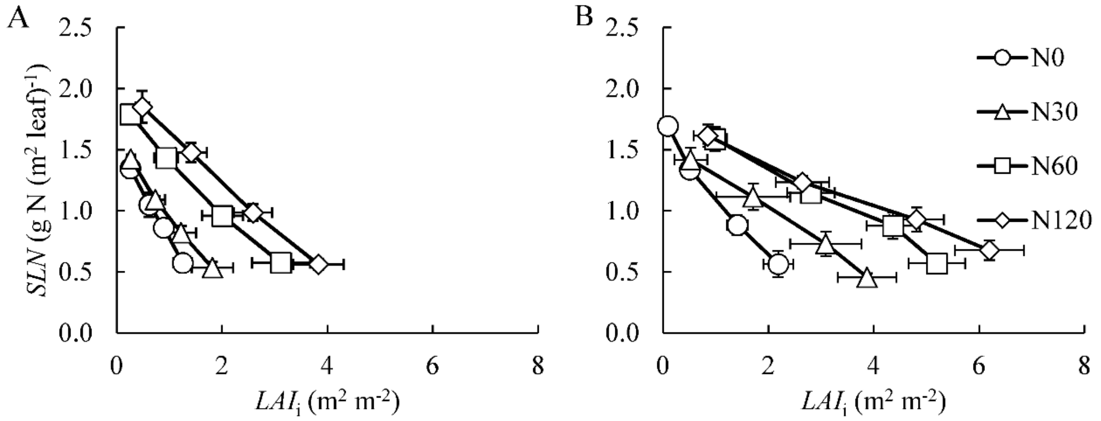

Supplementary Figure S2. Specific leaf nitrogen (*SLN*) against the leaf area index at depth i measured from the top (LAI_i). Data presented was obtained at linear growth (on 17 June in Panel A) and at full flowering (on 23 July in Panel B) in 2015. N0, N30, N60, N120 denote nitrogen fertilization rate in 2015 at 0, 30, 60 and 120 kg N ha^{-1}, respectively.

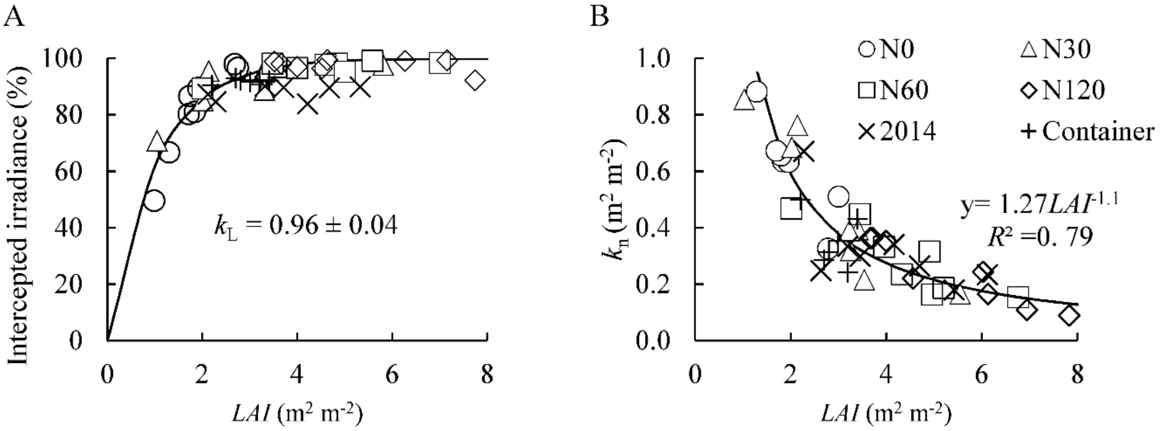

Supplementary Figure S3. Canopy light interception (Panel A) and nitrogen extinction coefficient (k_n; Panel B) against leaf area index (*LAI*) at different growth conditions. N0, N30, N60, N120 denote nitrogen fertilization rate in 2015 at 0, 30, 60, and 120 kg N ha^{-1}, respectively. “2014” denotes data collected in 2014 in the plots that received a nitrogen fertilization of 60 kg N ha^{-1}. Data collected in the other plots is not shown because there was severe weed competition. “Container” denotes data collected in the container experiment.

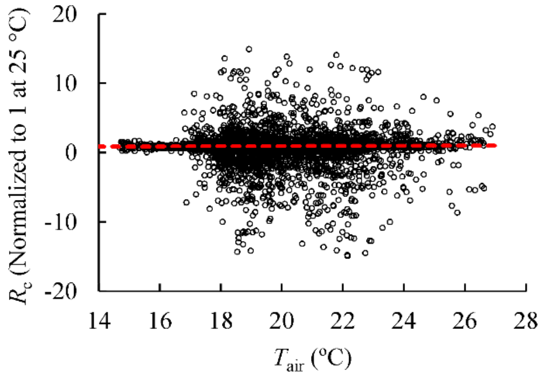

Supplementary Figure S4. Normalized canopy respiration (R_c) in relation to air temperature T_{air}.

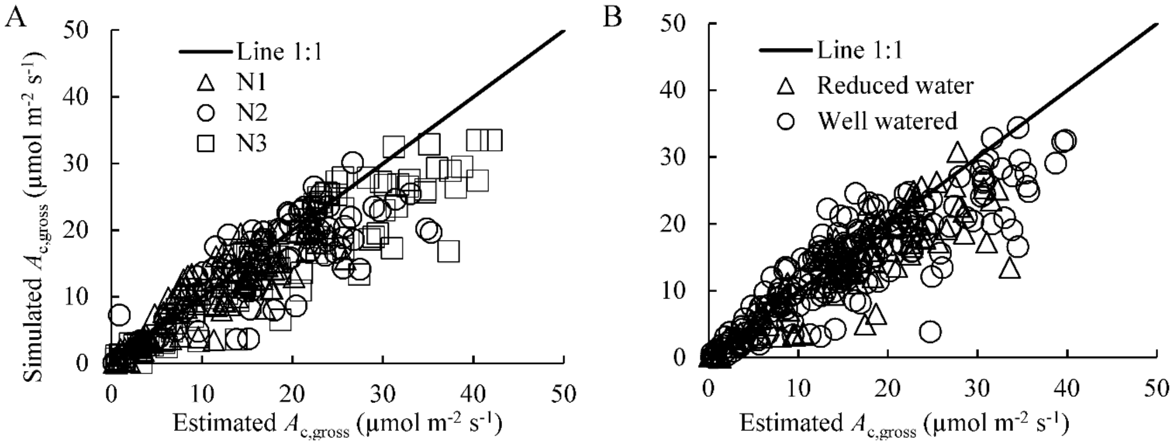

Supplementary Figure S5. Plots of simulated $A_{c,gross}$ against estimated $A_{c,gross}$ under different nitrogen and water regimes. Data presented were collected in CAN1. To avoid overcrowding of data points, only one tenth of the data is presented. N1, N2 and N3 denote the level of received nitrogen, see text for details.

Supplementary Table S1. List of parameters (± standard error, if applicable) of leaf photosynthesis model

Category	Symbol	Definition	Unit	Value	Reference
Leaf respiration	χ_{Rd}	Slope of linear relationship between R_{d25} and (SLN-n_b) [a]	µmol s^{-1} (g N)$^{-1}$	1.06 ± 0.08	Tang et al., 2017b
	E_{Rd}	Activation energy for R_d	J mol^{-1}	21634 ± 4085	Tang et al., 2017b
e^- transport	χ_{Jmax}	Slope of linear relationship between J_{max25} and (SLN-n_b) [a]	µmol s^{-1} (g N)$^{-1}$	220.7 ± 11.2	Tang et al., 2017b
	E_{Jmax}	Activation energy for J_{max}	J mol^{-1}	67292 ± 35986	Tang et al., 2017b
	D_{Jmax}	Deactivation energy for J_{max}	J mol^{-1}	114701 ± 28710	Tang et al., 2017b
	S_{Jmax}	Entropy term for J_{max}	J K^{-1} mol^{-1}	375 ± 82	Tang et al., 2017b
	κ_{2LL}	Efficiency of converting incident irradiance into linear electron transport (J) under limiting light	mol mol^{-1}	0.21 ± 0.004	Tang et al., 2017b
	θ	Convexity factor for the response of J to I_{inc}	-	0.70	Ögren & Evans, 1993
Enzyme kinetics and activity	χ_{Vcmax}	Slope of linear relationship between V_{cmax25} and (SLN-n_b) [a]	µmol s^{-1} (g N)$^{-1}$	101.3 ± 5.7	Tang et al., 2017b
	E_{Vcmax}	Activation energy for V_{cmax}	J mol^{-1}	63024 ± 1562	Tang et al., 2017b
	Γ^*_{25}	Γ^* at 25 °C	µmol mol^{-1}	37.5	Bernacchi et al., 2002
	$E_{\Gamma*}$	Activation energy for Γ^*	J mol^{-1}	24460	Bernacchi et al., 2002
	K_{mC25}	K_{mC} at 25 °C	µmol mol^{-1}	272	Bernacchi et al., 2002
	E_{Kmc}	Activation energy for K_{mC}	J mol^{-1}	80990	Bernacchi et al., 2002
	K_{mO25}	K_{mO} at 25 °C	mmol mol^{-1}	165	Bernacchi et al., 2002
	E_{KmO}	Activation energy for K_{mO}	J mol^{-1}	23720	Bernacchi et al., 2002
	χ_{Tp}	Slope of linear relationship between T_{p25} and (SLN-n_b) [a]	µmol s^{-1} (g N)$^{-1}$	9.3 ± 0.7	Tang et al., 2017b
	E_{Tp}	Activation energy for T_p	J mol^{-1}	34417 ± 5298	Tang et al., 2017b
CO_2 diffusion	g_{s0}	Minimum stomatal conductance	mol m^{-2} s^{-1}	0.11 ± 0.04[b]	
	a_1	Empirical constant for g_s response to VPD	-	1.13 ± 0.10[b]	
	b_1	Empirical constant for g_s response to VPD	kPa^{-1}	0.42 ± 0.03[b]	
	E_{b1}	Activation energy for b_1	J mol^{-1}	-51755 ± 6389.4[b]	
	r_m:r_s	Ratio of mesophyll: stomatal resistances	-	1.05[b]	

[a]: SLN denotes specific leaf nitrogen, with which photosynthetically active leaf nitrogen is defined as $SLN-n_b$; n_b was assumed to be the SLN of senesced leaves, measured at 0.25 g N m^{-2} in this study.

[b]: the value was derived using the data collected in Tang et al. (2017b) for the purpose of the present study.
